# The Octarepeat Region of the Prion Protein Is Conformationally Altered in PrP^Sc^

**DOI:** 10.1371/journal.pone.0009316

**Published:** 2010-02-24

**Authors:** Alice Y. Yam, Carol Man Gao, Xuemei Wang, Ping Wu, David Peretz

**Affiliations:** Research & Development, Novartis Vaccines & Diagnostics, Inc., Emeryville, California, United States of America; Ohio State University, United States of America

## Abstract

**Background:**

Prion diseases are fatal neurodegenerative disorders characterized by misfolding and aggregation of the normal prion protein PrP^C^. Little is known about the details of the structural rearrangement of physiological PrP^C^ into a still-elusive disease-associated conformation termed PrP^Sc^. Increasing evidence suggests that the amino-terminal octapeptide sequences of PrP (huPrP, residues 59–89), though not essential, play a role in modulating prion replication and disease presentation.

**Methodology/Principal Findings:**

Here, we report that trypsin digestion of PrP^Sc^ from variant and sporadic human CJD results in a disease-specific trypsin-resistant PrP^Sc^ fragment including amino acids ∼49–231, thus preserving important epitopes such as the octapeptide domain for biochemical examination. Our immunodetection analyses reveal that several epitopes buried in this region of PrP^Sc^ are exposed in PrP^C^.

**Conclusions/Significance:**

We conclude that the octapeptide region undergoes a previously unrecognized conformational transition in the formation of PrP^Sc^. This phenomenon may be relevant to the mechanism by which the amino terminus of PrP^C^ participates in PrP^Sc^ conversion, and may also be exploited for diagnostic purposes.

## Introduction

Prion diseases are fatal neurodegenerative disorders characterized by dementia, motor dysfunction, and spongiform degeneration of the brain [1]. Propagation of the infectious prion particle is attributable to a conformational conversion of the widely expressed normal prion protein PrP^C^ into an abnormal, infectious conformation termed PrP^Sc^. PrP^Sc^, unlike the physiological protein PrP^C^, exists predominantly in an aggregated form and is partially resistant to protease digestion [2]. Digestion with Proteinase K (PK) leaves behind a core particle termed PrP^res^ (for resistant PrP) or PrP^27–30^ (for 27–30 kDa PK-resistant fragments) that consists of the carboxy-terminal two-thirds of the protein. Consequently, the PK-labile amino-terminus has been suggested to be solvent-accessible and largely unstructured in the context of PrP^Sc^ as it is in PrP^C^ [3], [4]. Furthermore, because PrP^27–30^ remains infectious and because of the scarcity of tools to isolate full-length PrP^Sc^, the amino-terminus has remained relatively unexamined in the context of aggregated PrP.

The amino-terminal tail of PrP is known to contain an array of five almost identical octapeptide sequences, also termed octarepeats, that has been reported to play a role in copper binding and homeostasis [5], as well as in protection from oxidative stress (reviewed in [6]). Importantly, mutations that result in expansion of the octarepeats have been linked to familial Creutzfeldt-Jakob Disease (CJD). Documented cases of familial CJD report insertions of 2–9 octapeptide sequences [7]–[9], whose impact are recapitulated in transgenic mouse models [10]. Likewise, transgenic mice expressing PrP that lack all five octapeptide sequences appear to be impaired in propagating PrP^Sc^, as these mice have longer incubation periods before they become symptomatic, lower prion titers, reduced amounts of PrP^res^, and no observable histopathology [11], [12]. *In vitro* studies also support a role of the octarepeats in PrP^Sc^ replication as octapeptide insertions or deletions affect the rate and propensity of oligomerization for recombinant PrP [13], [14]. As such, understanding the function of the amino-terminal portion of PrP is critical for understanding propagation of prion diseases.

Despite the importance of the amino-terminus, previous prion research has primarily focused on PK-treated PrP^Sc^, partly because of the difficulties associated with separating PrP^Sc^ from PrP^C^ in infectious samples. Other biochemical means of protein enrichment such as antibody immunoprecipitation are largely ineffective at separating PrP^C^ from PrP^Sc^ due to their sequence identity. PK digestion has been employed to circumvent this issue, but at the cost of removing amino-terminal sequences and decreasing yields of PrP^Sc^ and scrapie-associated infectivity [15]. To address this issue, we tried employing the more specific enzyme trypsin, rather than PK, to distinguish PrP^C^ from PrP^Sc^. We found that indeed trypsin cleavage significantly digested PrP^C^, but retained the majority of PrP^Sc^, thus providing a means to separate the isoforms while maintaining the octarepeat sequence. Using this technique, we found the octarepeat sequence had multiple epitopes exposed in PrP^C^ but not PrP^Sc^, suggesting that there is a conformational transition in this region during the conversion of PrP^C^ to PrP^Sc^. Given the putative role of the octarepeat in infectivity, this novel structural change may provide clues to the mechanism of PrP^Sc^ replication.

## Results

### Trypsin Digests PrP^C^ While Preserving PrP^Sc^

**Figure 1:**
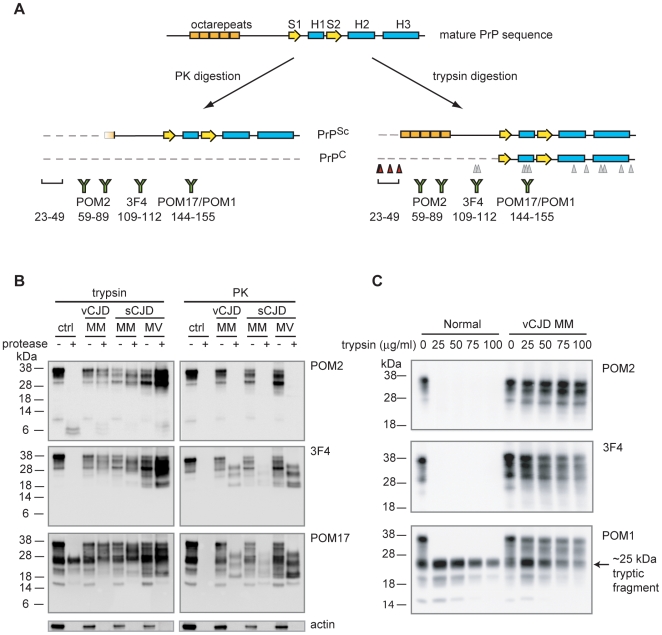
Trypsin significantly digests PrP^C^ while preserving almost full-length PrP^Sc^. *A.* Schematic of the full-length mature PrP sequence, PK-digested PrP^Sc^ and PrP^C^, and trypsin-digested PrP^Sc^ and PrP^C^. Trypsin cleavage sites are indicated by triangles, with gray triangles lying within the PK-resistant core and red triangles indicating expected cleavage sites. *B.* 50 µg/ml trypsin or PK was incubated with normal (ctrl), vCJD MM (codon 129 polymorphism), sCJD MM, and sCJD MV BHs. Samples were separated by SDS-PAGE and immunoblotted with POM2, 3F4, and POM17 antibodies. An actin immunoblot was performed as a proteolysis control. *C.* Trypsin removes the octarepeats and 3F4 epitope of PrP^C^, but not helix one. Normal and vCJD BHs were digested with 0, 25, 50, 75 or 100 µg/ml trypsin and then analyzed by immunoblotting with POM2, 3F4, and POM1 antibodies.

To characterize the physicochemical properties of the PrP^Sc^ octarepeat region, we needed to preserve amino-terminal sequences while still removing PrP^C^ from CJD samples. Given the nonspecific nature of PK digestion, we chose to digest the samples with a more specific enzyme, trypsin, which cleaves only on the C-terminal side of lysine and arginine residues. The predicted tryptic map for the human PrP sequence shows that the majority of cleavage sites are within the PK-resistant core (Fig. 1A, gray triangles) with a few additional cleavage sites between residues 23–49 of the mature PrP sequence (Fig. 1A, red triangles) and no sites within the octarepeat region (residues 51–91). To confirm this prediction, we digested brain homogenates (BHs) from several CJD strains in parallel with PK as a control. Immunoblot analysis of the digests indicated that trypsin-resistant PrP fragments (expected to be ∼49–231) had higher mass than PK-resistant fragments (∼90–231) with a substantial fraction of molecules preserved (Fig. 1B, 3F4 antibody recognizing residues ∼109–112 and POM17 antibody recognizing residues ∼144–155). Mass spectrometry of human recombinant PrP_23–231_ validated that trypsin could cleave at all the sites predicted by the PrP sequence, thus confirming that the tryptic sites were conformationally protected in PrP^Sc^ (data not shown). Further examination revealed that digestion with trypsin but not PK preserved the PrP octapeptide repeats recognized by the POM2 antibody (Fig. 1B, POM2 antibody recognizing residues ∼59–89). Importantly, preservation of this region significantly enhances PrP detection (Fig. S1).

By contrast, PrP^C^ was digested by both proteases, and was no longer detected by POM2 and 3F4 (Fig. 1B and 1C, Normal). However, when we immunoblotted the digests with POM17 or POM1 antibody (recognizing helix one, residues ∼144–155), we observed a trypsin-resistant fragment of ∼25 kDa. Given that the structure of PrP^C^ is composed of a globular domain (∼122–231) following an unstructured amino-terminus [19], [20], the 25 kDa trypsin-resistant fragment likely included the globular domain as it was the appropriate molecular weight and detected by POM1 and POM17 but not POM2 or 3F4 antibodies (Fig. 1B, 1C). Interestingly, this would suggest that the globular domain is tightly folded and that the tryptic sites within the POM17/POM1 epitope (R148 and R151) are not accessible for cleavage, contrary to how they appear in the solution structure [20]. To better understand the resistance of this domain to digestion, we digested both normal and variant CJD (vCJD) BHs with increasing amounts of trypsin (Fig. 1C). The amino-terminal sequences of PrP^C^ were extremely labile as no PrP was detected by either POM2 or 3F4 at any of the trypsin concentrations tested, but a PrP^C^ 25 kDa fragment and PrP^Sc^ from vCJD BH were resistant to digestion. A survey of other non-CJD BHs confirmed the presence of a 25 kDa PrP^C^ tryptic fragment, indicating that the fragment was not unique to that sample (data not shown). However, as most of our studies of PrP^Sc^ utilized 3F4 and POM2 epitopes, PrP^C^ trypsin-resistant fragments did not impact our analysis.

### Measuring the Stability of PrP^Sc^ Epitopes Against Trypsin Digestion

**Figure 2:**
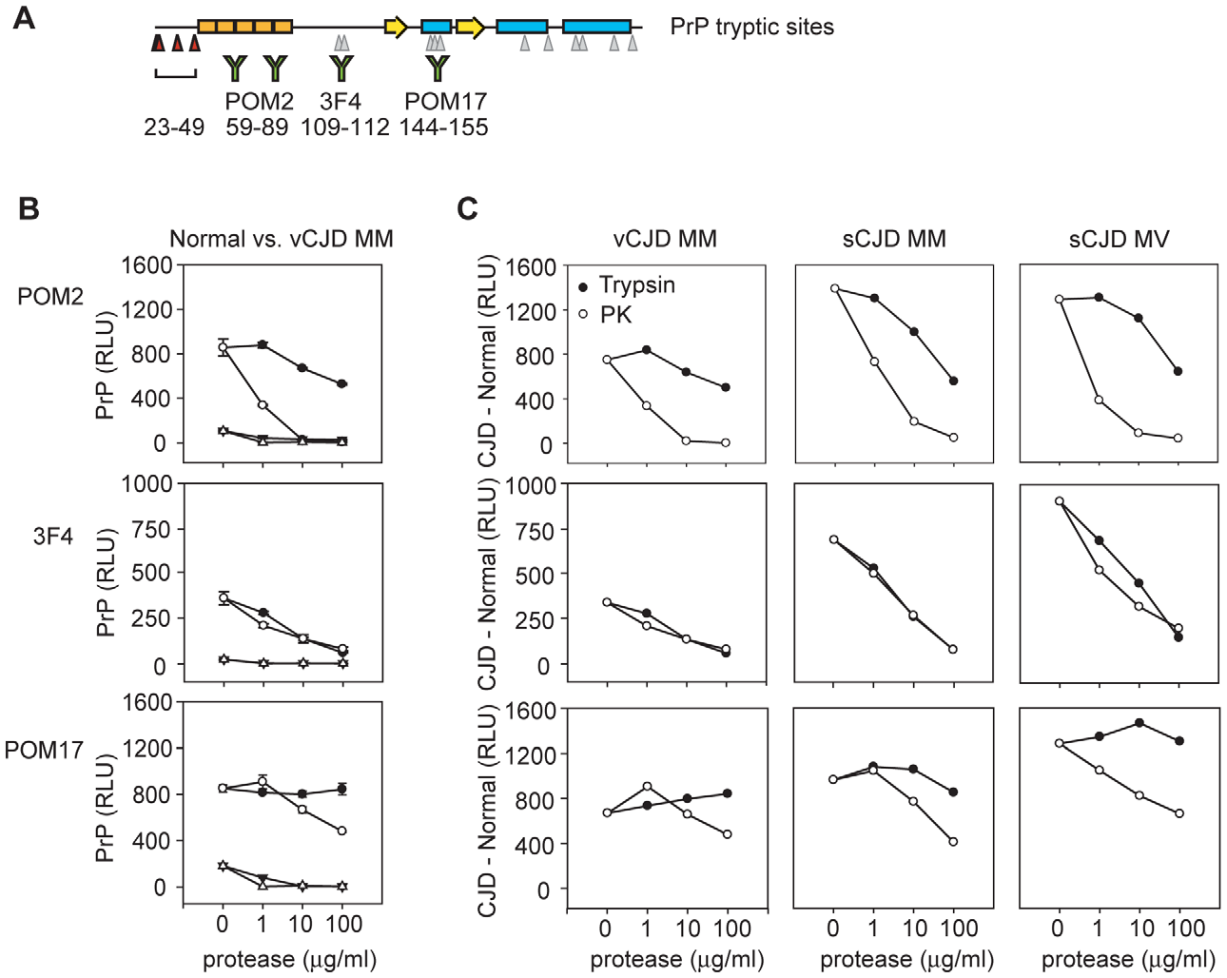
Sensitivity of PrP^Sc^ epitopes to trypsin digestion. *A.* Schematic of trypsin cleavage map of PrP with the indicated locations of antibody epitopes. *B & C.* Normal and CJD BHs were digested with increasing amounts of trypsin (filled symbols) or PK (open symbols). Digests were centrifuged, pellets were denatured, and PrP was detected by direct ELISA. *B.* Detection of digested normal (triangles) and vCJD (circles) samples is shown in relative light units (RLU). *C.* Detection of vCJD MM, sCJD MM, and sCJD MV samples digested with trypsin or PK are shown in a normalized format by subtracting away the signal derived from normal samples (CJD – Normal, RLU). Error bars in *B* indicate the standard deviation measured from samples prepared in triplicate. Similar errors were measured for CJD samples but are not shown for normalized data in *C*.

To better quantitate the sensitivities of specific PrP epitopes to PK or trypsin cleavage, we measured protease-resistant epitopes by direct ELISA (Fig. 2A). Normal (129 MM), vCJD (129 MM), and sporadic CJD (sCJD) (129 MM and 129MV) BHs were digested with increasing amounts of trypsin or PK, followed by centrifugation. PrP in the pellets was denatured, coated to microtiter plates, and detected by direct ELISA. The centrifugation protocol efficiently separated PrP^C^ from PrP^Sc^, with the small amount of residual PrP^C^ efficiently removed via proteolysis with 1 µg/ml protease (see Fig. 2B, compare PrP^C^ from normal BH and PrP^C^+PrP^Sc^ from vCJD digested BH). To directly compare the amount of PrP^Sc^ preserved by each protease, we subtracted the PrP^C^-derived signal detected in normal BH from each of the infectious samples (Fig. 2C, CJD - Normal).

As expected, we found significant differences in the exposure of PrP^Sc^ epitopes to trypsin versus PK digestion. Equal amounts of the core particle were preserved with either protease, as shown by 3F4 reactivity (Fig. 2C), whereas the octarepeat region was preserved only for trypsin digested PrP^Sc^ as shown by POM2 reactivity. The octarepeats were excised only at high concentrations of trypsin but rapidly disappeared with PK digestion (Fig. 2C, POM2). POM17 detection of the PrP^Sc^ core particle was consistent with our results via 3F4 detection and confirmed that the vast majority of helix one was resistant to trypsin digestion (Fig. 2C, POM17). On the other hand, POM17-detected PrP decreased with increasing PK despite the PK-resistance of the PrP^Sc^ core particle, in agreement with previous reports that high concentrations of PK will also digest the PK-resistant core (reviewed in [21]). Because PK is a nonspecific protease, PK may cleave at certain sites resulting in conformational destabilization of the PrP^Sc^ core particle and further proteolysis. It is likely that trypsin does not recognize these sites. Therefore, sequences such as the octarepeat and helix one regions are uniquely preserved in trypsin digestion, making trypsin an ideal tool for analysis of PrP^Sc^.

### The Octarepeat Region Undergoes a PrP^Sc^-Specific Conformational Change

**Figure 3:**
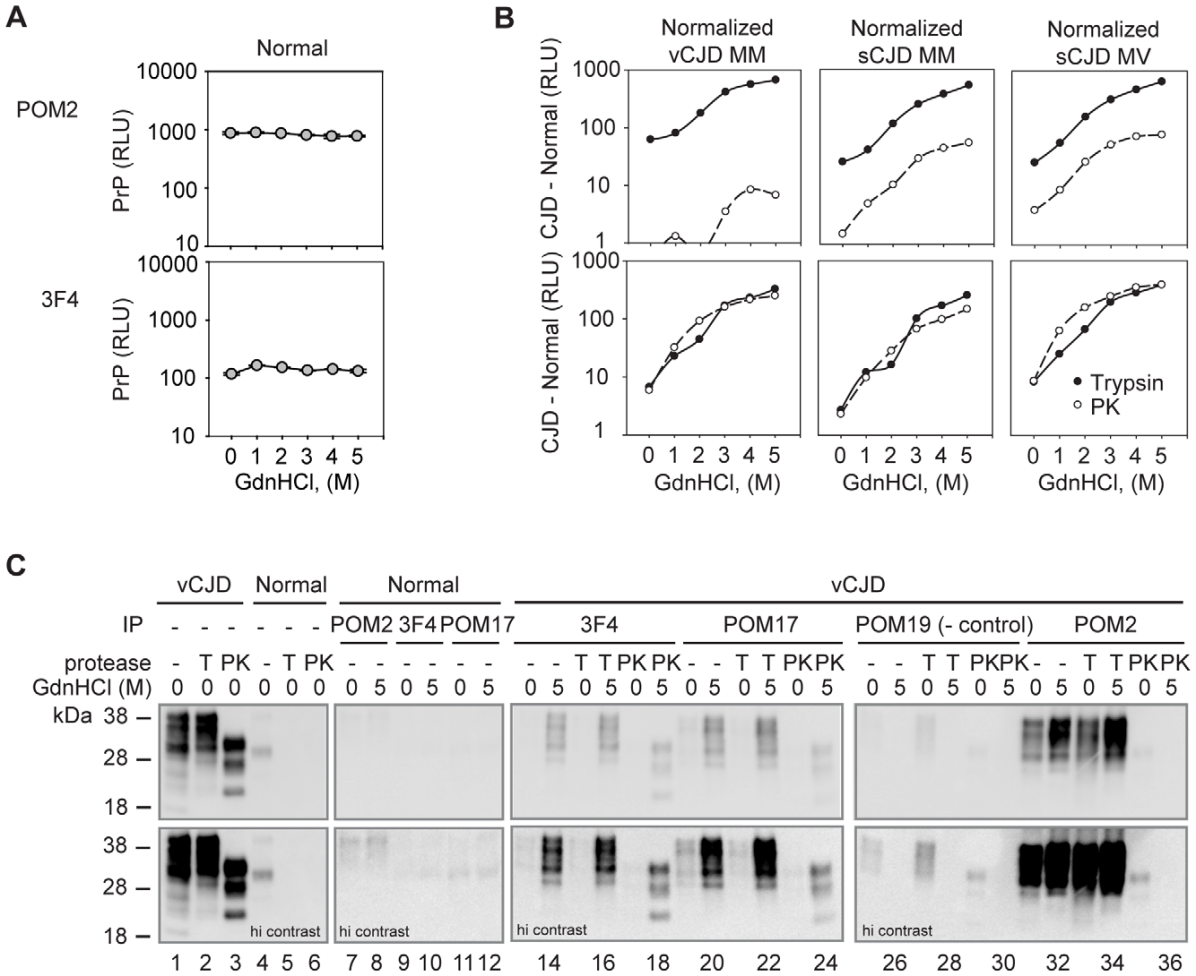
Epitope presentation of native and chemically denatured PrP^Sc^. *A.* PrP^C^ from normal BH was coated to ELISA plates, denatured with 0–5M GdnHCl, and detected by direct ELISA with the indicated antibodies. Results are shown in relative light units (RLU). *B.* Normal and CJD BHs treated with 50 µg/ml trypsin (filled symbols) or PK (open symbols) were centrifuged. Pellets were then coated to ELISA plates in duplicate, treated with 0–5 M GdnHCl, and PrP was detected by direct ELISA. The data shown is normalized by subtracting the signal derived from normal samples from that of the CJD samples (CJD – Normal, RLU). All plots are shown in log scale. *C.* PrP from vCJD or Normal BHs was digested and pelleted as in *B*, treated with 0 or 5 M GdnHCl, renatured, and immunoprecipitated with either a negative control (POM19) or with POM2, 3F4 or POM17 antibodies. Immunoprecipitations and 40% of the total immunoprecipitation input was immunoblotted with POM1 antibody. Error bars in *A* indicate the standard deviation measured from samples prepare in duplicate. Similar errors were measured for CJD samples in *B* with all coefficients of deviations less than 15% for signals above background. Errors are not shown for normalized data.

Despite extensive studies of PrP variants bearing mutations of the octarepeat region, understanding of the properties of this domain remain unclear. Preservation of the region by digestion with trypsin allowed us to study its structure by an epitope protection approach. Digested PrP^Sc^ from CJD BHs was coated directly to ELISA plates and probed with various antibodies to identify conformationally hidden epitopes. The stability of these conformations could be assessed by treatment with increasing amounts of denaturant (guanidine hydrochloride, GdnHCl) and looking for recovery of immunoreactivity. This ELISA-based approach allowed us to survey several different strains in both a high throughput and quantitative manner. Importantly, maximal antibody binding was observed for PrP^C^ at all concentrations of GdnHCl surveyed, demonstrating that the epitopes examined are accessible in the native conformation of PrP^C^ and that guanidine treatment of the passively-coated material did not strip PrP from the plate (Fig. 3A).

We next examined PrP^Sc^ from CJD brain homogenates. The high endogenous levels of PrP^C^ were digested away by PK or trypsin treatment and the immunoreactivity of PrP^Sc^ was tested after treatment with denaturant. Increasing amounts of GdnHCl yielded dramatic increases in 3F4 reactivity for all strains (Fig. 3B), consistent with previous reports that this region undergoes a major conformational change upon conversion of PrP^C^ to PrP^Sc^ [22]–[24].

Next, we examined the conformational stability of the octarepeat region which has been previously presumed to be unstructured in PrP^Sc^. Consistent with this view, significant POM2 immunoreactivity was observed even under native PrP^Sc^ conditions after trypsin digestion (Fig. 3B, POM2). However, when PrP^Sc^ was unfolded with GdnHCl there was a ∼5–10 fold increase in POM2 immunoreactivity, suggesting that this region indeed is buried in PrP^Sc^ and undergoes a conformational change upon conversion of PrP^C^ to PrP^Sc^. As expected, significantly different levels of PrP^Sc^ were detected after PK digestion. No POM2 signal was observed for PK-digested PrP^Sc^ from vCJD BH, in agreement with the complete digestion of the octapeptides in this strain [25], [26]. Many, if not all, sCJD isolates are reported to contain both type 1 and 2 PrP^Sc^ [27], [28], thus retaining a mixture of PrP^Sc^ protease-resistant fragments with some containing a single octapeptide (type 1) after PK digestion [25], that could be detected by POM2 (Fig. 3B, POM2 detection of sCJD). Interestingly, PK-resistant PrP containing a single octapeptide had a similar conformational stability as the trypsin-digested PrP which retains all octarepeats (Fig. 3B, compare POM2 curves for trypsin and PK).

We further confirmed these findings by immunoprecipitation studies for the vCJD sample, which appeared to contain the most homogeneous PrP^Sc^ by quantitative ELISA assays (Fig. 3C). Normal and vCJD BHs were digested, PrP^Sc^ was centrifuged (Fig. 3C, lanes 1–6, input), and PrP^Sc^ pellets were treated with or without denaturant, and immunoprecipitated with antibodies recognizing the various epitopes of interest. The immunoprecipitates were then examined by POM1 immunoblot (Fig. 3C, lanes 13–36). As expected, antibodies with epitopes buried within the protease-resistant core (3F4 and POM17) immunoprecipitated denatured but not native PrP^Sc^ relative to negative controls (Fig. 3C, POM19 immunoprecipitations; POM19 does not recognize human PrP).

Next, we examined the ability of POM2 to bind the octapeptide repeats. Consistent with our ELISA studies (Fig. 3B), no PrP was immunoprecipitated from PK-digested PrP^Sc^ (Fig. 3C). On the other hand, PrP^Sc^ from trypsin-digested BHs could be immunoprecipitated in agreement with the finding that one or more of the octapeptides are accessible (Fig. 3C). Similar to what we observed by ELISA, a larger proportion of PrP could be immunoprecipitated via POM2 antibody when PrP^Sc^ was first denatured with guanidine treatment. Thus, the increased yield of POM2 immunoprecipitation upon denaturation of PrP^Sc^ supports the idea that the octapeptide domain undergoes a structural transition upon formation of PrP^Sc^.

## Discussion

### A Structural Change in the Octarepeat Region of PrP^Sc^ and Prion Propagation

Despite extensive debate, the importance of the PrP octapeptide sequences in PrP^Sc^ pathophysiology remains unclear. Initially, the region was thought to be unnecessary since PK-digested PrP^Sc^ from tissue samples still proved to be infectious. However, emerging evidence suggests that the octapeptide sequences may play a role in propagating prions. For instance, insertion of 1–9 octapeptides into the PrP sequence has been linked to several cases of familial CJD [9], [29], [30]. The effects of this octarepeat expansion have been recapitulated in transgenic mice carrying additional octapeptide sequences who demonstrate clinical (ataxia), histopathological (neuronal apoptosis and accumulation of aggregated PrP), and biochemical (protease-resistant PrP) hallmarks of prion disease [10]. While transgenic mice expressing PrP lacking the octapeptide sequences remain susceptible to scrapie infection, these mice appear to be impaired in PrP^Sc^ propagation as they exhibit longer incubation times, no histopathology, and significantly reduced prion titers and protease-resistant PrP [11]. *In vitro* aggregation studies also support involvement of the octarepeat region in PrP oligomerization. For instance, expansion of the octarepeats increased the rate of oligomerization for recombinant PrP [13], while deletion of the octarepeats resulted in reduced oligomerization [14]. These data together demonstrate that the octarepeat region is not required for disease progression, but that it may facilitate prion propagation and/or pathogenesis.

Given that the octarepeats are largely PK-sensitive, it has been presumed that the region contributes little structure to PrP^Sc^. Here, by using trypsin digestion of PrP^Sc^ to preserve most of the PrP sequence (estimated ∼49–231) we have revealed that there is indeed a structural transition in the octarepeat region in the conversion of PrP^C^ to PrP^Sc^. The structural change we measured upon denaturation (5–10 fold increase in detected PrP) mimics the structural changes observed within the PK-resistant core (Fig. 3B, 3F4) [22], [24], [31].

Several possibilities exist for how the octarepeat region may affect prion disease pathology. First, the octapeptide sequences may modulate the toxicity of PrP^Sc^. Currently, the loss of infectivity resulting from PK digestion is largely attributed to the digestion of some proportion of PrP^Sc^ (∼90–231) [15], [17], but it is possible that infectivity is lost with the PK-labile amino-terminus as well. Second, the octapeptide sequence may be prone to self-association and cause multimerization of PrP^C^, a process that, even if not strictly required for generation of prions, could accelerate conversion to PrP^Sc^. *In vitro* binding and aggregation studies with octapeptide multimers of varying lengths support this theory [32], [33]. Our finding that the octarepeats undergo a structural transition in the conversion of PrP^C^ to PrP^Sc^ open up another possibility that structural components of the octarepeat region, either alone or in conjunction with the primary sequence, may enhance the ability of PrP^Sc^ to propagate.

### PrP^Sc^ Isolation by Trypsin Digestion

We have developed a novel method of purifying PrP^Sc^ from infectious samples that preserves the majority of the PrP sequence. Future studies comparing the infectious titers of PK- and trypsin-digested samples would allow the contribution of the octarepeats to infectivity to be directly measured. This method could also be used as an enhanced diagnostic tool. Our sandwich ELISA was found to yield superior detection by preserving multiple POM2 epitopes. Because its cognate epitope is repeated on PrP, POM2 can dock to PrP through multiple interactions, yielding extremely high avidities in the femtomolar range [18]. This peculiarity renders trypsinization, followed by ELISA, one of the most sensitive methods for detecting authentic PrP^Sc^ (Fig. S1, Methods and Results S1). The sensitivity gained by additional POM2 epitopes is also apparent in the amount of PrP captured by POM2 immunoprecipitation (Fig. 3C). Chemical denaturation of PrP exposed additional epitopes, enhancing the amount of PrP captured several fold over a single POM2, 3F4 or POM17 epitope. In addition, since PK-digested PrP^Sc^ has a high propensity to fibrillize in solution [34], preservation of full-length PrP^Sc^ may enhance the solubility of the aggregate, making it more amenable to structural studies – e.g. by solid-state nuclear magnetic resonance spectroscopy which necessitates large amounts of highly purified analytes. Moving forward, we now have the ability to purify PrP^Sc^ that closely approaches unmodified, physiological aggregates. This new tool may help to dissect the role of the octarepeat region in prion disease.

## Materials and Methods

### Tissue Samples

CJD brain homogenates (BHs) were acquired from the NIBSC CJD Resource Centre and correspond to variant CJD (vCJD) MM (NHBY0/0014: in Figures 1B, 2–3, and S1), vCJD MM (NHBY0/0003: in Figure 1C only), sporadic CJD (sCJD) MM (NHBX0/0001), and sCJD MV (NHBX0/0004) strains with the indicated reference codes and genotypes at polymorphic residue 129 [16] (http://www.nibsc.ac.uk/spotlight/cjd/brainsamples.html). Normal BH was retrieved from the tissue bank of the Swiss National Reference Centre of Prion Diseases (Zürich, Switzerland). All 10% BHs (w/v) were homogenized in 0.25 M sucrose. Protein concentrations were measured by BCA Assay (Pierce, Rockford, IL) with most 10% BHs being ∼10 mg/ml.

### Antibodies

POM2, POM17, POM1, and POM19 mouse monoclonal antibodies were obtained from the laboratory of Dr. Adriano Aguzzi at the Institute of Pathology at the University Hospital of Zürich. POM2 recognizes the sequence QPHGG(G/S)W whereas POM17 and POM1 recognize the helix one region of PrP (∼144–155) and compete with 6H4 antibodies for binding [17], [18]. POM19 recognizes residues 121–134 and 218–221 of the murine PrP sequence, but does not recognize the human PrP sequence. 3F4 (Covance, Princeton, NJ) recognizes residues ∼109–112 (MKHM) in the human PrP sequence. Anti-actin antibodies (clone C4, Millipore, Billerica, MA) recognize residues ∼50–70.

### Western Blot Analysis

25 µg of normal and infectious BH (∼2.5 µl of 10% BH) was digested with 50 µg/ml of trypsin or Proteinase K (PK) in 0.5×TBS (25 mM Tris pH 7.5, 75 mM NaCl) with 0.5% Tween20, 0.5% TritonX-100, and 5 mM CaCl_2_ for 1 hr at 37°C. Samples were separated by 12% SDS-PAGE in parallel with 10 µg undigested sample, and immunoblotted with anti-PrP antibodies (3F4, POM2, POM17, or POM1) followed by an HRP-conjugated goat anti-mouse polyclonal antibody (Pierce). Chemiluminescent images were acquired via a Kodak Image Station 4000MM.

### ELISA

10% BH was diluted 10-fold into TBS with 2% Sarkosyl (TBSS) and digested with 0, 1, 10, or 100 µg/ml trypsin or PK for 1 hr at 37°C. Digestions were stopped by adding 2 mM PMSF and Complete Mini protease inhibitor cocktail (Roche, Indianapolis, IN) in four volumes of TBS. The samples were then detected by direct ELISA. Briefly, approximately 500 nl of digested 10% BH was centrifuged at 14,000 rpm for 30 minutes at 4°C. The PrP^Sc^ pellets were denatured in 6M GdnSCN, diluted with an equal volume of 0.1M NaHCO_3_ pH 8.9, and passively coated to ELISA plates overnight. The plates were washed and blocked in 0.1× BlockerCasein in TBS (Pierce) and coated PrP was detected via 0.1 µg/ml 3F4, POM2 or POM17 antibodies and an alkaline phosphatase (AP)-conjugated goat anti-mouse polyclonal antibody (Pierce). Samples were analyzed by ELISA in triplicate and washed six times with TBS 0.05% Tween20 between antibody incubations. Finally, LumiphosPlus substrate (Lumigen, Southfield, MI) with 0.05% SDS was added to the wells and incubated for 30 minutes at 37°C before the luminescence was measured via a Luminoskan luminometer (Thermo Electron Corporation, Waltham, MA).

### Epitope Exposure of PrP^Sc^

10% BH diluted 10-fold into TBSS was digested with 50 µg/ml trypsin or PK for 1 hr at 37°C. Digestions were halted and samples were centrifuged as described above. PrP^Sc^ pellets were resuspended in 0.1M NaHCO_3_ pH 8.9, and passively coated to ELISA plates overnight. The next day, coated proteins were denatured with increasing concentrations of GdnHCl (0–5M) for 15 minutes at 37°C, and detected by direct ELISA using 0.5 µg/ml 3F4 or 0.1 µg/ml POM2 antibodies as described above.

### Immunoprecipitation of Native or Denatured PrP^Sc^

10% BH diluted 10-fold into TBSS was digested with 50 µg/ml trypsin, PK, or nothing for 1 hr at 37°C. Digestions were halted and samples were centrifuged as described above. PrP^Sc^ pellets were then resuspended in 0 or 5M GdnHCl, and adjusted to a final concentration of 0.1M GdnHCl in TBS with 1% TritonX-100 and 1% Tween20. PrP was subsequently immunoprecipitated with POM2, 3F4, or POM17-conjugated to Protein G Dynal beads and analyzed by POM1 immunoblot. (PrP^Sc^ from ∼75 µg BH was precipitated with ∼10 µg antibody.) POM19 immunoprecipitations served as a negative control as POM19 does not recognize human PrP. Approximately 40% of an equivalent sample was loaded to assess the input for each immunoprecipitation.

## Supporting Information

Figure S1 Preservation of the octapeptide sequences allows enhanced detection of PrP. Normal or vCJD BHs were digested with increasing concentrations of trypsin (closed circles) or PK (open circles) and detected by sandwich ELISA. Samples were captured on 3F4-coated plates and detected with either alkaline phosphatase-conjugated POM2 or POM17 antibody (RLU). The signal contributed by PrPSc was estimated by subtracting Normal BH-derived signal from vCJD BH-derived signal.
(0.45 MB TIF)

Methods and Results S1 (0.03 MB DOC)
