# Supplementary figures and images for: The Octarepeat Region of the Prion Protein Is Conformationally Altered in PrPSc
Source: PLoS One. 2010 Feb 24;5(2):e9316. doi: 10.1371/journal.pone.0009316 (PMC2827544; doi:10.1371/journal.pone.0009316)

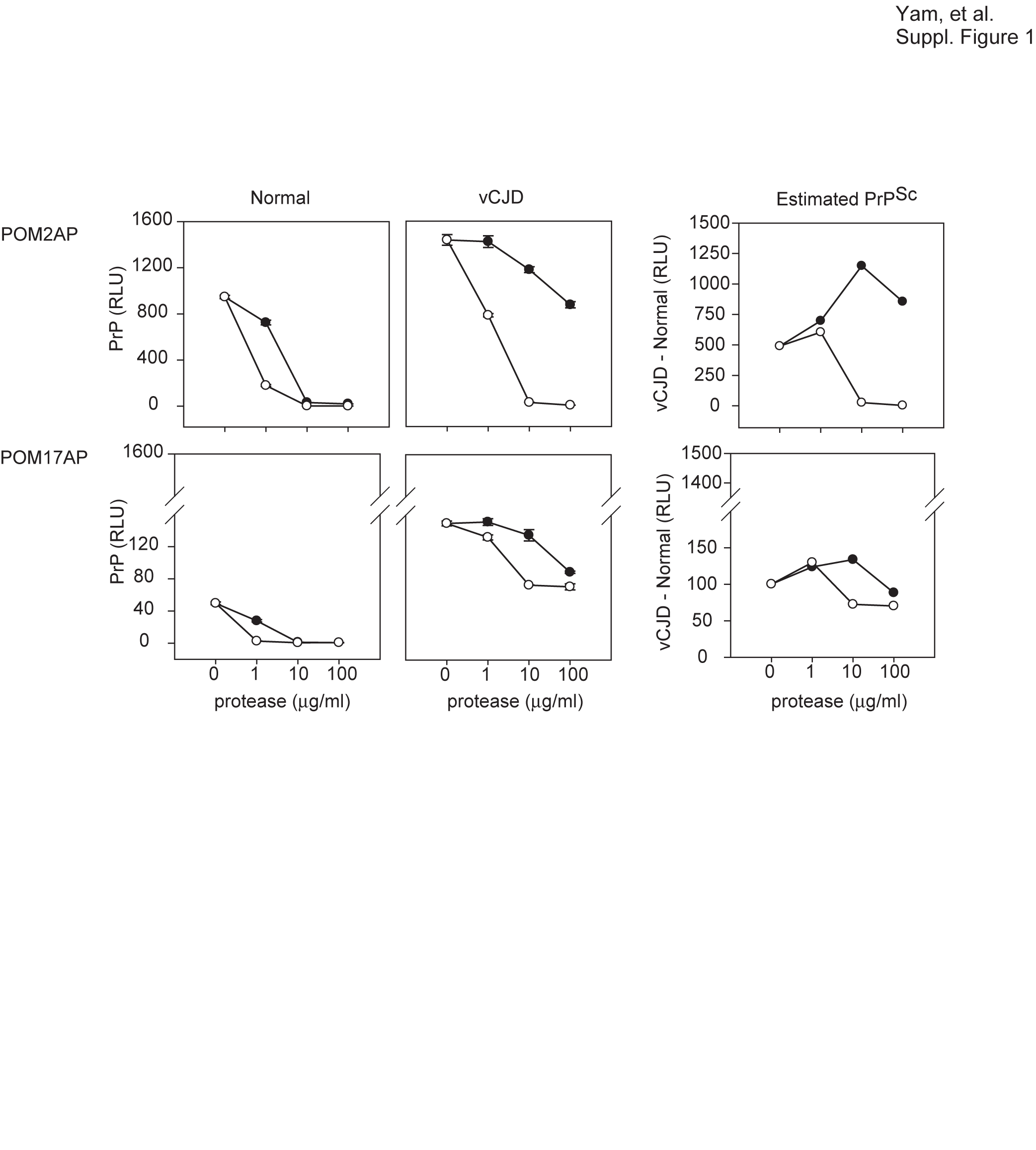

Supplement: Figure S1 — Preservation of the octapeptide sequences allows enhanced detection of PrP. Normal or vCJD BHs were digested with increasing concentrations of trypsin (closed circles) or PK (open circles) and detected by sandwich ELISA. Samples were captured on 3F4-coated plates and detected with either alkaline phosphatase-conjugated POM2 or POM17 antibody (RLU). The signal contributed by PrPSc was estimated by subtracting Normal BH-derived signal from vCJD BH-derived signal. (0.45 MB TIF) [file pone.0009316.s001.tif]
