# Supplementary material for: The Octarepeat Region of the Prion Protein Is Conformationally Altered in PrPSc
Source: PLoS One. 2010 Feb 24;5(2):e9316. doi: 10.1371/journal.pone.0009316 (PMC2827544; doi:10.1371/journal.pone.0009316)
Supplement: Methods and Results S1 — (0.03 MB DOC) [file pone.0009316.s002.doc]

# METHODS

10% BH diluted 10-fold into TBS with 2% Sarkosyl (TBSS) was digested with 0, 1, 10, or 100 μg/ml trypsin or PK for 1 hr at 37°C. Digestions were stopped by adding 2 mM PMSF and Complete Mini protease inhibitor cocktail (Roche, Indianapolis, IN) in four volumes of TBS. The samples were then detected by sandwich ELISA. Briefly, 250 nL of digested 10% BH was denatured, reconditioned, and PrP was captured by 3F4-coated plates (375 ng/well) and detected with 0.02 μg/ml AP-conjugated POM2 or POM17. All samples were analyzed by ELISA in triplicate and were washed six times with TBS 0.05% Tween20 between antibody incubations. Finally, LumiphosPlus substrate (Lumigen, Southfield, MI) with an enhancing solution was added to the wells and incubated for 30 minutes at 37°C before the luminescence was measured via a Luminoskan luminometer (Thermo Electron Corporation, Waltham, MA).

### RESULTS

### Preservation of the octarepeats allows enhanced detection of PrP^Sc^

Our experience with PrP immunoassays suggested that antibodies recognizing the octarepeats often confer enhanced detection, probably due to the increased number of antibody epitopes as well as to the avidity effect that generates an extremely high affinity interaction between the antibody and its epitope. As such, we asked whether we could attain higher detection levels of PrP^Sc^ by virtue of preserving the octarepeat region via trypsin digestion. Accordingly, we digested normal and infectious brain homogenates with increasing amounts of either trypsin or PK and detected the remaining PrP by sandwich ELISA using 3F4 as a capture antibody followed by detection with alkaline phosphatase-conjugated POM2 or POM17 (Fig. S1). Of note, the PrP^C^-derived signal in both the normal and infectious BHs decreased with increasing protease when detected with either POM2 or POM17. The ~25 kDa trypsin-resistant fragment recognized by POM17 was not detected since this tryptic fragment does not retain the 3F4 epitope and is eliminated in the initial capture step of the sandwich ELISA with 3F4. For both variant (Fig. S1) and sporadic (data not shown) CJD strains, POM17-detected PrP was comparable for trypsin or PK-digested samples, although trypsin preserved more epitopes at certain protease concentrations. By contrast, POM2-detected PrP^Sc^ rapidly disappeared with increasing amounts of PK in the digestion, but decreased much more slowly with increasing amounts of trypsin. Furthermore, detection by POM2 was enhanced 10-fold over detection via POM17, a result that is attributable to the avidity of the POM2 antibody for the repeated octapeptide sequence.
